# Survey Methods of the 2018 International Tobacco Control (ITC) Japan Survey

**DOI:** 10.3390/ijerph17072598

**Published:** 2020-04-10

**Authors:** Mary E. Thompson, Christian Boudreau, Anne C.K. Quah, Janine Ouimet, Grace Li, Mi Yan, Yumiko Mochizuki, Itsuro Yoshimi, Geoffrey T. Fong

**Affiliations:** 1 Department of Statistics and Actuarial Science, University of Waterloo, Waterloo, ON N2L 3G1, Canada; cboudreau@uwaterloo.ca; 2 Department of Psychology, University of Waterloo, Waterloo, ON N2L 3G1, Canada; ackquah@uwaterloo.ca (A.C.K.Q.); j2ouimet@uwaterloo.ca (J.O.); y47li@uwaterloo.ca (G.L.); mi.yan@uwaterloo.ca (M.Y.); geoffrey.fong@uwaterloo.ca (G.T.F.); 3 Division of Tobacco Policy Research, National Cancer Center Japan, 5-1-1 Tsukiji, Chuo-ku, Tokyo 104-0045, Japan; mochizuki@jcancer.jp; 4 Japan Cancer Society, 13th Floor, Yurakucho Center Bldg. 2-5-1, Yurakucho, Chiyoda-ku, Tokyo 100-0006, Japan; iyoshimi@ncc.go.jp; 5 School of Public Health and Health Systems, University of Waterloo, 200 University Ave W., Waterloo, ON N2L 3G1, Canada; 6 Ontario Institute for Cancer Research, 661 University Ave Suite 510, Toronto, ON M5G 0A3, Canada

**Keywords:** Japan, tobacco control policy, survey weights, response rate, cooperation rate

## Abstract

This paper describes the methods of the Wave 1 (2018) International Tobacco Control (ITC) Japan Survey. The respondents were adults aged 20 years and older in one of four user groups: (1) cigarette-only smokers who smoked at least monthly and used heated tobacco products (HTPs) not at all or less than weekly, (2) HTP-only users who used HTPs at least weekly and smoked cigarettes not at all or less than monthly, (3) cigarette-HTP dual users who smoked at least monthly and used HTPs at least weekly, and (4) non-users who had never smoked or who smoked less than monthly and used HTPs less than weekly. Eligible respondents were recruited by a commercial survey firm from its online panel. Respondents were allocated proportionally to sample strata based on demographic, geographic, and user type specifications benchmarked to a national reference. Survey weights, accounting for smoking/HTP use status, sex, age, education, and geography, were calibrated to benchmarks from a nationally representative survey in Japan. Response rate was 45.1% and cooperation rate was 96.3%. The total sample size was 4615 (3288 cigarette smokers, 164 exclusive HTP users, 549 cigarette-HTP dual users, and 614 non-users). The 2018 ITC Japan Survey sampling design and survey data collection methods will allow analyses to examine prospectively the use of cigarettes and HTPs in Japan and factors associated with the use of both products and of transitions between them.

## 1. Introduction

This paper describes Wave 1 (3 February–2 March 2018) of the International Tobacco Control (ITC) Japan Survey. The ITC Japan Survey was designed to collect survey data from Japanese cigarette smokers, heated tobacco product (HTP) users, dual cigarette-HTP users and non-users regarding their knowledge, attitudes, beliefs, perceptions, behaviors, and use patterns associated with cigarette smoking and HTP use.

The paper briefly outlines the tobacco control policies of Japan and the key objectives of the ITC Japan Survey, the sampling design, survey data collection, outcome rates, and the approach used for weighting data in an effort to generate a sample broadly representative of Japan’s population of adult cigarette smokers, HTP users, dual users of cigarettes and HTPs, and non-users when the data were collected. The paper also summarizes the strengths and weaknesses of the ITC Japan Survey methodology. A full description of the survey methods of the ITC Japan Survey is available in the Wave 1 ITC Japan Technical Report [1].

## 2. Tobacco Control Policy Impact in Japan

As of February 2020, a total of 180 countries have ratified the World Health Organization (WHO) Framework Convention on Tobacco Control (FCTC). This includes Japan, which ratified the FCTC in June 2004, thereby obligating its government to implement strong tobacco control policies. These include the WHO FCTC Guidelines [2]: higher taxes to reduce demand (Article 6), comprehensive smoke-free laws (Article 8), large pictorial health warnings (Article 11), comprehensive measures to eliminate or limit tobacco advertising, promotion, and sponsorship (Article 13), and support for cessation (Article 14). Japan has not yet implemented any FCTC policies at the highest level, likely due to the Japanese government’s 30% stake in Japan Tobacco [3,4]. Japan has weak smoke-free laws [5]: smoking is allowed in most indoor public venues; several cities have enacted stronger restrictions on smoking outdoors (e.g., no street smoking) [4]. The strongest of Japan’s tobacco control policies—taxation—falls below the WHO recommendation of 70% tax share of price [6] (the current retail price of cigarettes is 63% [7]). However, in October 2018, Japan launched a plan to increase cigarette taxes by 1 yen per stick over the next four years [8]. This policy over the coming years will be critical, as increasing cigarette taxes is accepted as the most effective measure for reducing smoking prevalence [9,10,11]. There have been very few empirical studies that examined the population impact of Japan’s partial implementation of FCTC policies, including implementation of partial smoke-free laws, and allowing only 30% text-only warnings as well as misleading descriptors such as light/mild. Several studies in Japan have examined factors and reasons for smoking or quitting [12,13], while others have studied nicotine dependence [14], cultural norms [15], economic disadvantage [16], gender [17], and cessation support [18,19]. The absence of comprehensive studies of tobacco use and the effects of tobacco control policies in Japan remains a gap in the literature.

## 3. Objectives of the ITC Japan Survey

The terminology describing alternative nicotine delivery devices is evolving and may include some degree of ambiguity across studies. Heated tobacco products (HTPs) refer to products containing tobacco in sticks, pods, or capsules that are heated in an electronic device with a heating element. HTPs include actual solid tobacco that is heated at a lower temperature (~350 °C) than combustible cigarettes [20]. Thus, HTPs are a sub-category of the broader class of alternative nicotine delivery products (ANDS), which includes another sub-category of liquid-only vaping devices, also known as e-cigarettes. Although the liquid in vaping devices may (and often does) contain nicotine, the distinction is that HTPs contain solid tobacco and vaping devices/e-cigarettes do not. HTPs are also distinct from combusted tobacco/ordinary cigarettes in that they do not strictly involve burning.

Although HTPs are not a new concept (they were introduced by the tobacco industry in the late 1980s), the new generation of HTPs, which includes IQOS (manufactured by Philip Morris International), Ploom TECH (by Japan Tobacco), and glo (by British American Tobacco), have been well-received in some markets, but are more prevalent in Japan than in any other country. IQOS was introduced in Japan in 2016; by September 2018, IQOS had captured 15.5% of the tobacco market, with the total HTP market share increasing to 21.4%, making Japan the epicenter for HTPs [21]. At the same time, cigarette sales plummeted—by 12.5% in 2017 and an additional 11.7% in 2018. Because of the rapid evolution and increased market share of HTPs in Japan, which are marketed as reduced-harm tobacco products, accompanied by the unprecedented decline in cigarette sales, the effects of HTPs on smoking cessation, uptake, and/or sustained use need to be better understood.

The ITC Japan Survey was designed to fulfill two broad objectives. The first was to examine the interplay between cigarettes and HTPs; in particular: (1) to portray how the use of tobacco differs for cigarette-only smokers and HTP-only users (some of whom may also be former cigarette smokers) both over time and between important subgroups (defined by age, sex, income, level of intention to quit cigarettes, nicotine dependence level); (2) to examine tobacco-use patterns and behavior and opinions associated with smoking among adults aged 20 and older. The second objective was to examine the effectiveness of key policies of the WHO Framework Convention on Tobacco Control (FCTC), the global tobacco control treaty.

## 4. ITC Japan Survey Measures

The ITC Japan Survey included many established measures used in ITC surveys in 28 other countries to examine the level of effectiveness of each of the FCTC policy domains over time, and in comparison to other ITC countries. The Wave 1 (2018) ITC Japan Survey questionnaire was developed to assess the research objectives as well as measure other constructs necessary to meet the survey objectives.

## 5. Sample Sizes, User Groups, and Quotas

The overall target sample size for the Wave 1 ITC Japan Survey was 4250 Japanese adults aged 20 or older and was divided into 4 user groups: cigarette-only smokers, HTP-only users, dual users of cigarette and HTP, and non-users. The focus is on the population aged 20 and older because both smoking and buying tobacco under the age of 20 are illegal in Japan. Table 1 provides the user type definitions and their respective target and actual achieved sub-sample sizes. The sub-sample targets were determined to ensure sufficient statistical precision and power to best achieve the research objectives.

Rakuten Insight first recruited cigarette-only smokers, which allowed the 3 other user-groups to populate concurrently; and then subsequently recruited for any remaining open targets. The achieved/final sample size was 4615, shown in Table 2. For the HTP-only user group, the sample was recruited based on sex allocated proportionally to Japanese benchmark data [22,23]. For the dual and non-user groups, the sample was recruited based on age and sex allocation; and for the cigarette-only smoker group, the sample was recruited based on age, sex, and geographic allocation. In all cases, targets were assigned to ensure that sub-samples were as representative as possible with regard to sex (all 4 user groups), age (all groups except HTP-only users), and geographic region (exclusive cigarette smokers).

## 6. Sample Source, Inclusion Criteria, Remuneration, and Survey Development

Members of the online panel in Japan of the commercial survey firm, Rakuten Insight, are recruited via a double opt-in process with email verification and deduplication. Registrants are recruited from a variety of online advertising channels, online affiliates, banner ads, e-commerce sites, etc. Panel profiles are updated annually by Rakuten Insight, and show the panel proportions to match census proportions on region and sex. The panel has substantial representation from each age decade from 20 to 59, while under-representing the 60+ age group. Based on this and other data quality checks, Rakuten Insight’s panel includes substantial numbers from all regions and demographic categories of the general population. Furthermore, the Rakuten Insight panel is the largest panel in Japan with over 2.5 million participants, about 2.4% of the total 20+ population [1].

Rakuten Insight used standard profile and demographic information provided by their panelists to identify and invite them to participate in the survey. Once invited to the survey, panelists first completed screening to ensure they met the following inclusion criteria: (1) aged 20 or older and (2) met one of the four user type definitions specified in Table 1 [1]. Panelists who qualified and completed the Wave 1 ITC Japan Survey were provided a standard number of points (based on the survey length) plus bonus points the equivalent to $3 USD upon submitting their survey data [1].

The survey content and logic were developed by the research team, consisting of international tobacco control and survey design experts, through a structured and iterative process of consultation and revision [1]. Survey content was developed in English and then translated by a professional English–Japanese translator into Japanese [1]. The Japanese translation was subsequently reviewed the Japanese research team. The survey specifications were then refined to ensure survey logic, question wording and response options, and all other survey elements were refined and cross-referenced for consistency, clarity, and accuracy [1].

The survey protocols and all materials, including the survey questionnaires, received ethics clearance from the Office of Research Ethics, University of Waterloo, Canada (ORE#31428). All participants provided consent to participate.

## 7. External Data Sharing

Data from the Wave 1 (2018) ITC Japan Survey are available to approved external researchers two years after the cleaned and weighted data sets were released by the ITC Data Management Centre at the University of Waterloo. Researchers who are interested in analyzing these data will be required to apply for approval by submitting a request to the International Tobacco Control Data Repository (ITCDR). Further information about the data sharing policies can be found on the ITC Project website [24].

## 8. Quality Control

Rakuten Insight released the initial invitations to their panel at deliberate intervals, and the survey activity was closely monitored to ensure that the data collection and related aspects were working correctly. This ‘soft launch’ or pilot data collection was conducted from 3–13 February 2018. The pilot data were subsequently reviewed by Rakuten Insight and the research team. Throughout the data collection phase, Rakuten Insight implemented the research protocol to ensure a smooth survey process and made available a portal link to a report monitoring survey activity in real time. Weekly survey activity reports and an analysis of next steps with respect to the recruitment strategy were also provided.

There were two criteria used in order to flag poor-quality data: (1) Seconds per question (SecperQ) and (2) the percentage of responses that were either Refused or Don’t Know (%RDK) [1]. In the Wave 1 ITC Japan Survey, very extreme values occurred for both of these variables: SecperQ values of less than 1.7 s per question, which by published estimates does not allow time for even reading the question, and %RDK responses for more than 70% of the questions completed by the respondent [1].

Rakuten Insight and the research team data analysts conducted a systematic analysis of the survey data to determine the records meeting the established criteria for a poor-quality respondent. The strategy was to create a group of normal respondents by dropping all poor-quality respondents (those with very low SecperQ and/or high %RDK) and to use this identified group to calculate normal behavior ranges [1].

## 9. Survey Outcome Rates

The response rate is 46.7% and the cooperation rate is 96.8%. Information about the disposition codes and the computation of rates is reported in further detail in the ITC Japan Survey Technical Report [1]. The cooperation rate is defined as the number of completed interviews as a percentage of the number of those who entered the survey and proceeded as far as confirming their eligibility [1,25]. The response rate is defined as the number of completed interviews as a percentage of an estimated number who were invited to the survey at a time when their quota was ‘open’ and who were eligible to participate [1,25]. The estimated number in the denominator of the response rate was obtained from observed eligibility rates for those whose eligibility status is known, and the observed quota open rate for the survey as a whole. The cooperation and response rates are based respectively on the Cooperation Rate 4 (COOP4) and Response Rate 4 (RR4) from the American Association for Public Opinion Research (AAPOR) [25].

In the main part of the fieldwork, there were 28,406 potential respondents who received invitations, and of these, 13,692 logged in to start the survey. It is estimated that overall, about 10,626 of those invited (not just those who logged in to start) met eligibility criteria, including belonging to a group for which the quota had not been filled. The number of respondents before the quality control exclusions was 4958, and the final number of respondents is 4615.

## 10. Survey Weights

Eight sets of cross-sectional weights were constructed for the Wave 1 ITC Japan Survey [1]. All sets were computed to correct and adjust for sample misrepresentation by frame error (e.g., under-coverage), non-response and other biases, as well as to improve precision of estimates through the use of auxiliary information. The first set of weights, which is the basis for the remaining 7 sets, is the cross-sectional inflation weights. These weights were computed by dividing all respondents of the final data set into the 4 groups mentioned previously. Short-term quitters (i.e., quit cigarette smoking within the last two years) were classified into two separate groups: short-term quitters using HTP at least weekly and short-term quitters using HTP less than weekly or not at all, while long-term quitters were grouped with non-users for weight calculation purposes. The weight calibration was performed using data from the 2017 Japan Society and Tobacco Internet Study (JASTIS Study) [22,23]. These weights are designed to make respondents in each of the 4 groups representative (with respect to sex, age groups, education level, and geographic regions of respondents) of the corresponding population at the time Wave 1 data were collected. The other 7 sets of weights are simply subsets of the cross-sectional inflation weights for different user types; e.g., cigarette smokers, dual users, HTP users, recent quitters, all tobacco users, non-users, and long-term quitters. For these 7 sets, the weights have been rescaled to sum to sample size to facilitate comparisons with other ITC countries. Information and guidance on which set of weights should be used can be found in the ITC Japan Survey Technical Report [1].

## 11. Limitations

Because of the rapidly evolving tobacco market in Japan, it was desirable to calibrate the survey weights to estimates of the user group sizes from a nearly contemporaneous nationally representative survey, namely the JASTIS Study. JASTIS included respondents aged up to 69 years, whereas the Wave 1 ITC Japan Survey included respondents older than 69 years. The JASTIS Study Profile [22,23] lists other strengths and limitations of the study; the latter include small sample sizes for HTP only users and recent quitters (both HTP and non-HTP users).

## 12. Conclusions

The Wave 1 ITC Japan Survey uses a sampling design and data collection method, together with an analytical strategy, intended to account for departures from the ideal situation of data from respondents recruited by probability sampling. As indicated in Table 1, this survey has yielded a baseline data set that captures many key behaviors, beliefs, attitudes, perceptions of tobacco products; reports of the impact of tobacco control policies, including all of the major policies of the WHO FCTC; and also other important characteristics that are relevant in explaining and understanding tobacco product use and transitions in product use of tobacco users and non-users in Japan in 2018. Because the ITC Japan Survey is a longitudinal cohort study, the measures of Wave 1, together with data from subsequent waves, establish a foundation for prospective analyses to address the study objectives: to measure and understand the impact of policy and non-policy factors on the use of cigarettes and HTPs and to understand patterns of transitions in product use—at each timepoint, and over time.

## Figures and Tables

**Table 1:** International Tobacco Control (ITC) Japan Survey Measures.

Demographics: Gender, Ethnicity, Age, Education, Income, State of Health
Other personal moderators: Quitting history, nicotine dependence, levels of stress, including financial stress, depressed mood, use of intoxicants (e.g., alcohol), time perspective, etc.
Environmental moderators: Number of smokers/users in the household and in social network
Policy-Specific (proximal) measures of policies on heated tobacco products (HTPs) and of WHO Framework Convention on Tobacco Control (FCTC) policies:
(a) Article 6: Price paid per unit of product, total weekly cost, product type/variant, purchasing unit, price perceptions. (b) Article 14: Use of cessation services and recall of advice, use of HTPs and/or other medicine use in conjunction with professional assistance, advice on appropriateness of HTP use. (c) Article 13: Advertising/marketing: noticing ads and frequency in key channels (TV, print, internet), susceptibility to advertising, whether HTP advertising makes them think about cigarettes. (d) Article 11: Health warnings: salience and noticing of health warnings (if any), brand usage, perceived risks, perceived impact on product use; forgoing cigarettes because of the warnings. (e) Article 8: Smoke-free/Vapor-free laws (and/or establishment policies): exposure to smoking/vaping in key venues, perceived impact on product use, reports on restrictions. (f) Product availability: Restrictions on access: perceived availability. (g) Article 9: Nicotine content, flavor and other product characteristics: nicotine content and flavors of HTP brands used, perceived addictiveness of HTPs and cigarettes, and appeal of HTPs. (h) Article 12: Awareness and recall of media campaigns on HTPs and on anti-smoking themes.
Psychosocial Mediator (distal) variables: Social norms for both vaporized nicotine products (VNPs) and cigarettes, outcome expectancies for products, reasons for VNP use, self-efficacy and intentions to quit smoking; relative harmfulness, health concerns, functions of smoking, substitutability of functions to VNP.
VNP and Tobacco use behaviors: Key outcomes along with some of the distal variables for intermediary analyses. Use of VNPs and other tobacco products: frequency of use, duration, and intensity of use (e.g., cigarettes per day); usual brand/type of product; quit attempts (smoking), duration of abstinence (smoking), product switching.

**Table 2:** ITC Japan Wave 1 Survey sample sizes and definition of the user types.

User Groups	Definition	Targeted	Achieved
Cigarette-only smokers	Smokes cigarettes at least monthly and uses HTPs not at all or less than weekly	3000	3288
HTP-only users	Smokes cigarettes not at all or less than monthly and uses HTP at least weekly	200	164
Cigarette-HTP dual users	Smokes cigarettes at least monthly and uses HTPs at least weekly	450	549
Never and non-users	Smokes cigarettes not at all or less than monthly and uses HTPs not at all or less than weekly	600	614
**Total**		**4250**	**4615**
